# Association between Vitamin Intake during Pregnancy and Risk of Small for Gestational Age

**DOI:** 10.3390/nu9121277

**Published:** 2017-11-23

**Authors:** Inmaculada Salcedo-Bellido, Juan Miguel Martínez-Galiano, Rocío Olmedo-Requena, Juan Mozas-Moreno, Aurora Bueno-Cavanillas, Jose J. Jimenez-Moleon, Miguel Delgado-Rodríguez

**Affiliations:** 1 Department of Preventive Medicine and Public Health, School of Medicine, University of Granada, 18016 Granada, Spain; isalcedo@ugr.es (I.S.-B.); rocioolmedo@ugr.es (R.O.-R.); abueno@ugr.es (A.B.-C.); jjmoleon@ugr.es (J.J.J.-M.); 2 Ciber de Epidemiología y Salud Pública CIBERESP, 28029 Madrid, Spain; jmozasm@sego.es (J.M.-M.); mdelgado@ujaen.es (M.D.-R.); 3 Instituto de Investigación Biosanitaria de Granada ibs.GRANADA, Complejo Hospitalario Universitario de Granada/Universidad de Granada, 18012 Granada, Spain; 4 Department of Nursing, University of Jaen, 23071 Jaen, Spain; 5 Public Health System of Andalusia, 23400 Jaen, Spain; 6 University of Jaén, Campus de Las Lagunillas s/n, Building B3 Office 413, 23071 Jaén, Spain; 7 Obstetric and Gynecology Unit, Virgen de las Nieves Universitary Hospital, 18014 Granada, Spain; 8 Division of Preventive Medicine, University of Jaen, 23071 Jaen, Spain

**Keywords:** maternal nutrition, dietary vitamins intake, pregnancy, small for gestational age

## Abstract

Pregnancy increases the requirements of certain nutrients, such as vitamins, to provide nutrition for the newborn. The aim was to analyze the association between dietary intake of vitamins during pregnancy and risk of having a small for gestational age (SGA) newborn. A matched case-control study was conducted (518 cases and 518 controls of pregnant women) in Spain. Dietary vitamin intake during pregnancy was assessed using a validated food frequency questionnaire, categorized into quintiles. Odds ratios (ORs) and their 95% confidence intervals (CI) were estimated with conditional regression logistic models. A protective association was observed between maternal dietary intake of vitamins A and D and SGA. For vitamin B3 and B6, the observed protective effect was maintained after adjusting for potential confounding factors. For vitamin B9, we found only an effect in quintiles 3 and 4 (OR = 0.64; 95% CI, 0.41–1.00; OR = 0.58; 95% CI, 0.37–0.91). Protective effect for vitamin B12 was observed in 4th and 5th quintiles (OR = 0.61; 95% CI, 0.39–0.95; OR = 0.68; 95% CI, 0.43–1.04). No associations were detected between dietary intake of vitamins B2, E and C intake and SGA. Our results suggest a positive association between dietary vitamin intake during pregnancy and the weight of the newborn, although more studies are necessary and there could be a ceiling effect for higher intakes of some vitamins cannot be discarded.

## 1. Introduction

Low birth weight (LBW), which is defined as an infant’s birth weight ≤2500 g, is the major determinant of perinatal morbidity and mortality [1]. Worldwide, between 15% and 20% of all births are LBW, representing more than 20 million births a year; moreover, the majority occur in low- and middle-income countries, particularly in the most vulnerable populations [2]. However, birthweight depends directly on the gestational age and sex of the newborn. For this reason, gestational age should be considered when evaluating factors related to weight at birth; accordingly, it is usual to work directly with the concept of small for gestational age infant (SGA).

Maternal risk factors associated with an increased risk of SGA can be classified into: preconception factors (including socio-demographic characteristics such as marital and socioeconomic status and chronic diseases such as chronic hypertension), and pregnancy related conditions (including inadequate prenatal care and maternal risky behaviors such as maternal smoking, alcohol intake, other drug use or inadequate diet patterns for example) [3,4,5,6,7,8]. SGA also affects long-term health; respiratory infections, diabetes mellitus, obesity, heart disease as well as neuropsychiatric disorders have been reported among children with antecedent of SGA [9].

Several lifestyle factors also have a clear impact on SGA. Among these, dietary habits (potentially modifiable during pregnancy) may play an important role in its etiology as diet composition negatively impacts fetoplacental growth and metabolic patterns. In fact, poor maternal nutritional intake during pregnancy can also negatively impact the fetal genetic growth trajectory and can result in fetal growth restriction [10,11,12]. Pregnancy increases the requirements of certain nutrients due to the need to provide nutrition for the newborn [13]; for example, vitamins are essential elements for a mother’s normal metabolism as well as for fetal development. For example, vitamin A is required for maintaining adequate growth and development [14]. Deficient vitamin D status influences birth weight, SGA risk and neonatal growth [15], as does the vitamin B complex. Few studies investigate the relationship between dietary vitamin intake and SGA risk and they report contradictory results. In general, this relationship is addressed through the consumption of multivitamin/mineral supplements [16,17,18,19,20]. Moreover, most have been conducted in low- and middle-income countries [21,22,23]. These studies also show significant disparity in the reference time point: some studies take serum samples and cord blood after birth [14,24,25] and some use direct information on the first, second and third gestation term [21,23,26,27,28]. For these reasons, we aim to analyze the association between dietary intake of vitamins during pregnancy and risk of having a SGA newborn in Spanish pregnant women.

## 2. Material and Methods

We conducted a matched case-control study. The study population included women who gave birth to a singleton newborn in one of five hospitals of Eastern Andalusia (Spain): the University of Jaen Hospital (UJH), Ubeda Hospital (UH), the University of Granada Hospitals (two centers) (UGH), and Poniente Hospital (PH), serving a total of 1.8 million people. Case and control groups were collected from 15 May 2012 to 15 July 2015. Ethical Approval for this study was given by the Ethics Committees of the hospitals. All women included in the study signed an informed consent.

### 2.1. Cases

Eligibility criteria for cases were delivery of a single live newborn diagnosed as SGA, according to the tables developed for the Spanish population [29], without congenital malformations during the study period, and resident in the referral area of the hospital. Nineteen women declined to participate. A total of 533 cases were selected from the public hospitals: 79 (UJH), 46 (UH), 369 (UGH), and 39 (PH).

### 2.2. Controls

A match pair by maternal age at delivery (±2 years) was selected within the week following inclusion of a case in the same hospital. Eligible women were those having a non SGA newborn with the same inclusion criteria for cases (residence in the referral area of the hospital and no congenital malformations). Sixty-five women declined participation.

### 2.3. Data Collection

Three sources of data were used: (1) personal interviews (carried out within two days after delivery); (2) clinical charts; and (3) prenatal care records. Information was obtained on the following variables: mother’s demographic data (marital status, education level, age at the beginning of the pregnancy, ethnicity, , socioeconomic class, and occupation), obstetric history (parity and antecedent abortions, previous adverse perinatal outcomes), conditions during pregnancy (infections, preeclampsia, diabetes, and other obstetric conditions), smoking during pregnancy, prescribed and over-the-counter drugs, prenatal care (number of visits and date of first visit), and birth weight (weight in grams in the delivery room). Social class was classified based on the classification of the Spanish Society of Epidemiology ranging from I (the highest) to V (the lowest): class I (managerial and senior technical staff and freelance professionals); II (intermediate occupations and managers in commerce); III (skilled non-manual workers); IV (skilled manual workers); and V (unskilled manual workers) [30]. This classification is similar to that of the Black Report (document published by the Department of Health in the United Kingdom, which was the report of the expert committee into health inequality) [31]. Prenatal care utilization was measured by using the Kessner index (principal adequacy of prenatal care utilization index in use in the United States today) includes information about both the timing of prenatal care initiation and prenatal care visits after initiation [32].

Alcohol consumption before and during pregnancy was assessed by a structured questionnaire in which number of drinks and type of drink on weekdays, weekends (including Friday evening), and holidays (including the eve) were recorded.

### 2.4. Dietary Assessment

The baseline questionnaire included a semi-quantitative food frequency questionnaire (FFQ) previously validated in Spanish women aged 18–74 years, with 136 items and open-label questions for information about use of dietary supplements [33]. The questionnaire was based on typical portion sizes and had nine options for the frequency of intake in the previous year for each food item (ranging from never or almost never to ≥6 times/day). A dietitian updated the nutrient data bank using the latest available information recorded in the food composition tables for Spain [34,35]. Nutrient scores were computed using ad hoc computer software specifically developed for this purpose (for each food item, frequency by nutrient composition of specified portion size). After computing total energy intake. 15 matched pairs were excluded because of unreliable dietary assessment (total energy intake above 4000 Kcal/day), leaving 518 pairs for analysis (518 cases; 518 controls).

### 2.5. Statistical Analysis

Food and nutrient intakes were adjusted for total energy intake using the residuals method for cases and controls as is recommended by Willet et al. [36]. Energy-adjusted food or nutrient intakes were categorized in quintiles. Analyses were performed using Stata 13 (College Station, TX, USA). Odds ratios (ORs) and their 95% confidence intervals (CI) were estimated with conditional regression logistic models, with adjustment for potential confounders such as energy intake, preeclampsia, education level, pre-pregnancy body mass index (BMI), smoking, weight gain per week during pregnancy and previous preterm/LBW newborn. BMI was calculated as weight (in kg) just before pregnancy divided by height (in m) squared. Both weight and height were obtained from medical records of the women if possible, and self reported if not. All *p* values are 2-tailed. Statistical significance was set at *p* < 0.05.

## 3. Results

Table 1 summarizes the baseline characteristics of women included in the analysis. The weight gain during pregnancy and body mass index prior to gestation was higher among women in the control group women (*p* < 0.001). Regarding marital status, there were more married women in controls than in cases (*p* < 0.036). Classical risk factors such as previous preterm/LBW, smoking during the pregnancy and preeclampsia were more frequent in women with an SGA infant (*p* < 0.001). Intrauterine growth retardation was higher for cases compared to controls (*p* < 0.001).

Associations between dietary vitamin intake during pregnancy, categorized in quintiles, with risk of SGA are shown in Table 2 (vitamins A, D and E) and Table 3 (vitamins C and B complex) according to their lipophilic or hydrophilic character, respectively. Results from the adjusted analysis provide evidence of a protective association between maternal dietary intake of vitamins A and D and SGA, and no association for vitamin E (Table 2). For vitamin D, there was a decreased risk of SGA with its intake with the risk 38% less for quintile 5 compared with quintile 1.

Table 3 shows the level of intake of hydrosoluble vitamins in the case and control groups. No association was detected between vitamin C and SGA. With respect to B complex vitamins, excluding vitamin B2 (riboflavin), we found a protective effect that was not constant across quintiles and did not follow a clear trend for the different elements. Vitamin B1 was associated with a protective effect in quintiles 2, 4 and 5 in the crude analysis. This association disappeared after adjusting for potential confounding variables. However, with grouping quintiles 2 to 5 for vitamin B1, the net effect was an independent reduction of risk (ORa = 0.67; 95% CI, 0.48–0.94). For vitamin B3, a protective effect was observed in the crude analysis and this protective effect was maintained after adjusting for potential confounding factors.

A similar effect was observed for vitamin B6. There was a protective effect for quintiles 3 and 5. The global effect of quintiles 3 to 5 versus 1 to 2 after controlling for potential confounding factors was 0.75 (95% CI, 0.57–0.99). For vitamin B9 (folic acid), we found an effect for quintiles 2, 3 and 4, but only the latter two maintain an observed effect in multivariable models. In this case, pooling strata 2 to 5 versus quintile 1 had a protective effect (Ora = 0.70; 95% CI, 0.50–0.99). Finally, we found a protective effect for vitamin B12 in the 4th and 5th quintiles (ORa = 0.61; 95% CI, 0.39–0.95; Ora = 0.68; 95% CI, 0.43–1.04) (Table 3).

In our study population, with a measured multivitamin/mineral supplements consumption of 22.8% and 23.6%, respectively, of cases and controls, we did not find an effect of multivitamin/mineral supplements on SGA (ORa = 0.93; 95% CI, 0.66–1.29) (see Supplementary Table S1).

## 4. Discussion

We found an association between the intake of different vitamins and the risk of SGA. Vitamins D, B1, B3 and B12 identified a protective effect for higher intakes, for vitamins A, B6 and B9 moderate intakes was associated with a lower risk of SGA, and, no associations between dietary intake of vitamins B2, E or C and SGA were found.

Inconsistent results in previous studies show significant disparity in terms of nutrients assessed, methods used to evaluate diet and its nutrients (dietary intake: food frequency questionnaires, dietary record or prospective dietary record; or serum measurements), epidemiologic study design, prospective or retrospective collection of information, study population, sample size and setting (high, middle or low income countries), and also in the effect studied: mean weight at birth, percentage of low weight or SGA. These differences make the comparison of the results more difficult.

### 4.1. Interpretation and Clinical Significance of Findings

#### 4.1.1. Vitamin A

We found a protective effect of vitamin A, but only for moderate intake. In a previous study by Rajasingam et al., no association with SGA was found, although this study lacked inclusion of a control group from the same populations and not all women consented to blood samples for measurements of biomarkers [37]. Studies by Agarwal et al. and Gebremedhin et al. measured serum retinol after delivery and did not observe significant differences; however, these studies were based on a sample size of convenience, had a significant loss to follow-up or had been carried out in middle- or low-income countries [14,38]. The existing significant disparity in terms of methods used to evaluate diet and its involved nutrients could imply the need of studies with measurement of vitamin A levels to assess the possible contribution of poor vitamin A status to duration of pregnancy. Studies with well-defined objectives, adequate design, good methods of measuring the study variables, and enough study power are needed to make a correct approach to this problem.

Watson and McDonald observed that high β-carotene and retinol intakes were associated with decreased birthweight in a prospective cohort study of 439 European and Polynesian pregnant women assessing diet by a 24-h recall and a 3-day food record [39]. In addition, Al-Qaisi et al. observed that maternal vitamin A levels were significantly lower when offspring were LBW in a cohort of 88 pregnant Iraqi women [40].

#### 4.1.2. Vitamin D

For vitamin D, variable results have been reported. Studies in Western populations that did not find any association with SGA include Baker et al, and Carmago et al., who used intake data. However, these studies had difficulty in maintaining contact with participants or had a population sample from Massachusetts complicating the generalizability of these associations to regions with more sunlight exposure [26,41]. Rodriguez et al. and Morgan et al. [25,42] used biological markers, but were unable to obtain samples from all eligible participants introducing possible selection bias. Lastly, an association with low weight was not found by either Camargo et al. or Gale et al. based on dietary intake data and serum determinations [41,43].

Nonetheless, vitamin D is an essential nutrient with well-established roles in calcium metabolism [44], therefore, the positive effect we found is plausible. The majority of previous studies are consistent with our results. Based on serum determinations, several studies report that maternal vitamin D deficiency is associated with a higher frequency of SGA [15,21,45], or higher percentage of LBW [15,21,46]. Finally, studies based on dietary intake report a positive association with birth weight [39,47,48], and only Morgan’s study in Canada, reports a lower risk of LBW when levels of cord blood vitamin D are below 50 nmol/L [24].

#### 4.1.3. Vitamin E

Studies for vitamin E have described both a positive effect [22,49,50,51] and the absence of association [37,52]: a heterogeneity which is independent of the method used to evaluate the exposure and the level of development of the population studied. We found no association for vitamin E, probably due to the little impact it has on growth development as it has antioxidant properties fundamentally.

#### 4.1.4. Vitamin B Complex

Concerning the vitamin B complex, there are few studies in pregnancy investigating all B complex vitamins and most examine the effects of either riboflavin (B12) or folate (B9) alone or as part of a multivitamin/mineral supplement. In studies where multivitamin/mineral supplements are used, it is difficult to separate the effects of the individual B vitamins on the outcome studied. We have found studies that report a positive association for vitamin B2 [53], B9 [23,26,28], and B12 [23,28,39], while others have found an absence of effect for vitamin B9 [24,27,52,54] and B12 [24,26,54]. Our data, in contrast, show a protective effect for B9 and B12, both of which have an important role in supporting maternal and fetal health during pregnancy to build and preserve maternal stores and meet the needs of rapidly growing tissues [55] and increase the risk of neural tube defects and may contribute to preterm birth [54].

#### 4.1.5. Vitamin C

We did not find association between vitamin C and SGA which could be attributed to the fact that we studied a Mediterranean population with a high intake of fruits, mainly citrus, and vegetables, although there are studies that have found an association between Vitamin C and placental weight [37,53].

## 5. Multivitamin/Mineral Supplements

Recent systematic reviews report the ability of multivitamin/mineral supplements to decrease the risk of birth defects and the frequency of LBW [16,17]. However, the evidence supporting this comes principally from studies conducted in low- and middle-income countries, and suggests the effect is due to poor diet quality and diversity in poor resource settings [17]. Furthermore, these studies did not consider possible adverse effects of supplementation. The population of southern Spain is characterized by a high dietary diversity [56] so in principle the intake of multivitamin/mineral supplements is not necessary.

## 6. Strengths and Limitations

The effect of the intake of vitamins during pregnancy should not be assessed in isolation; the intake of vitamins rich food possibly has an additional role to the strictly metabolic effect of the vitamin and cannot be replaced by complex multivitamin [57].

The strengths of our study are: (1) our sample is representative of a reference population of around 12,000 healthy pregnant women attending the several Andalusian public hospitals; (2) we used previously utilized Spanish fetal growth curves to define adequate size for gestational age [29]; (3) we used a FFQ previously validated in the Spanish population [33,58] and previously used in Spanish pregnant women [59]; (4) the control group was selected by density in the same hospitals as cases and they represent the regular dietary vitamin intake of the reference pregnant female population from the area serviced by those hospitals; and (5) it is possible establish a suitable temporal relation.

We should also note that the absence of a clear dose-response gradient suggests either a threshold value below which the desired effect is not achieved or a saturation level above which greater intakes are not associated with a higher benefit. This makes it difficult to study the role played by vitamins on SGA and therefore these results should be further supported by a larger number of studies.

Our study also includes other limitations: (1) It was difficult to obtain exact information about our principal exposure variable, i.e., dietary vitamin intake during pregnancy. However, to decrease this limitation, we used a validated food frequency questionnaire [33,58]; (2) All questionnaires were recorded after birth but before hospital discharge, with the intention of estimating the average dietary intake during pregnancy. However, delivery and the last gestational week of pregnancy would be unlikely to change their habitual gestational dietary patterns; (3) The information was taken by midwives. This may introduce a classification bias as the participating women may want to respond with answers they believe will please the midwives: A bias that would affect both groups and shift the magnitude of the force of association toward the null value.

We cannot discard a memory bias, but if present, we think it will be a non-differential bias as beforehand no relation between a particular food intake and SGA is assumed. Finally, residual confounding cannot be completely ruled out.

## 7. Conclusions

In conclusion, a high consumption of vitamins D, B1, B3, and B12, and moderate intake of vitamins A, B6 and B9 during pregnancy were associated with lower risk of SGA. Our results suggest a positive association and a suitable temporal relation between dietary vitamin intake during pregnancy and weight of the newborn, although there could be a ceiling effect for higher intakes of some vitamins. From a public health point of view, it is desirable to reinforce maternal counseling and encourage dietary vitamin intake during pregnancy to decrease risk of SGA newborns and secondarily reduce its effects [1].

## Figures and Tables

**Table 1:** Description of the study population among healthy pregnant Spanish women for AGA (Adequate for Gestational Age) and SGA (*n* = 1036).

	**Cases(SGA)**		**Controls (AGA)**		***p* Value**
	**518**		**518**		
	***n***	**(%)**	***n***	**(%)**	
**Marital status**					0.036
Single	37	(7.1)	42	(8.1)	
Living with partner	161	(31.1)	124	(23.9)	
Married	320	(61.8)	352	(68.0)	
**Education level**					0.084
Primary	112	(21.6)	93	(17.9)	
High school—not finished	42	(8.1)	28	(5.4)	
High school	185	(35.7)	190	(36.7)	
University	179	(34.6)	207	(40.0)	
**Antecedent of preterm/Low Birth Weight (LBW) newborn**	64	(12.4)	26	(5.0)	<0.001
**Smoking during current pregnancy**	149	(28.8)	80	(15.4)	<0.001
**Preeclampsia**	46	(8.9)	11	(2.1)	<0.001
**Intrauterine growth retardation**	141	(27.2)	8	(1.5)	<0.001
**Kessner index (prenatal care)**					0.737
Adequate	259	(50.0)	253	(48.8)	
Intermediate	185	(35.7)	182	(35.2)	
Inadequate	74	(14.3)	83	(16.0)	
	**Cases (SGA)**		**Controls (AGA)**		***p* Value**
	**518**		**518**		
	**Mean**	**(SD)**	**Mean**	**(SD)**	
**Weight gain during pregnancy (g/week) mean (SD)**	278	(121)	310	(114)	<0.001
**Pre-pregnancy BMI mean (SD)**	23.1	(4.5)	23.9	(4.1)	<0.001
**Alcohol intake (g/week) mean (SD)**	4.2	(18.5)	3.1	(15.2)	0.312

SGA: Small for Gestational Age; AGA: Adequate for Gestational Age; SD: Standard Deviation

**Table 2:** Level of intake of liposoluble vitamins during pregnancy among healthy Spanish women (*n* = 1036).

	Cases (*n* = 518)		Controls (*n* = 518)		SGA ^a^			
	*n*	(%)	*n*	(%)	cOR ^b^	95% CI	aOR ^c^	95% CI
**Vitamin A** (mg/day) **(retinol)**								
Q1 (≤1.236)	135	(26.1)	104	(20.1)	1 (reference)		1 (reference)	
Q2 (1.237–1.545)	80	(15.4)	104	(20.1)	0.59 *	0.40–0.87	0.58 *	0.37–0.90
Q3 (1.546–1.979)	106	(20.5)	103	(19.9)	0.81	0.55–1.18	0.89	0.58–1.37
Q4 (1.979–2.604)	103	(19.9)	104	(20.1)	0.75	0.51–1.11	0.77	0.50–1.19
Q5 (>2.604)	94	(18.2)	103	(19.9)	0.69	0.47–1.01	0.67	0.43–1.05
**Vitamin D** (µg/day) **(calciferol)**								
Q1 (≤3.793)	112	(21.6)	104	(20.1)	1 (reference)		1 (reference)	
Q2 (3.794–5.168)	124	(23.9)	104	(20.1)	1.11	0.76–1.61	1.12	0.73–1.72
Q3 (5.168–6.228)	105	(20.3)	103	(19.9)	0.95	0.64–1.41	0.94	0.61–1.47
Q4 (6.229–7.981)	97	(18.7)	104	(20.1)	0.89	0.61–1.31	0.84	0.54–1.30
Q5 (>7.981)	80	(15.4)	103	(19.9)	0.73	0.49–1.08	0.62 *	0.40–0.98
**Vitamin E** (mg/day) **(α-tocopherol)**								
Q1 (≤3.793)	103	(19.9)	104	(20.1)	1 (reference)		1 (reference)	
Q2 (3.794–5.168)	103	(19.9)	104	(20.1)	1	0.67–1.49	1.03	0.66–1.63
Q3 (5.168–6.228)	101	(19.5)	103	(19.9)	0.99	0.66–1.47	1.18	0.75–1.84
Q4 (6.229–7.981)	85	(16.4)	104	(20.1)	0.81	0.54–1.23	0.85	0.54–1.35
Q5 (>7.981)	126	(24.3)	103	(19.9)	1.26	0.85–1.87	1.36	0.87–2.13

^a^ SGA: Small for Gestational Age; ^b^ cOR: Crude odds ratio and confidence intervals (95% CI) ^c^ aOR: Adjusted odds ratio by energy intake, preeclampsia, education level, pre-pregnancy body mass index, smoking, weight gain per week during pregnancy, and previous preterm/LBW newborn; * Association significant.

**Table 3:** Level of intake of hydrosoluble vitamins during pregnancy among healthy Spanish women (*n* = 1036).

	**Cases (*n* = 518)**		**Controls (*n* = 518)**		**SGA ^a^**			
	***n***	**(%)**	***n***	**(%)**	**cOR ^b^**	**95% CI**	**aOR ^c^**	**95% CI**
**Vitamin C** (mg/day) **(ascorbic acid)**								
Q1 (≤151.84)	121	(24.4)	104	(20.1)	1 (reference)		1 (reference)	
Q2 (151.85–201.05)	98	(18.9)	104	(20.1)	0.79	0.54–1.17	0.81	0.53–1.26
Q3 (201.06–253.52)	110	(21.2)	103	(19.9)	0.92	0.63–1.38	0.84	0.55–1.29
Q4 (253.53–322.23)	96	(18.5)	104	(20.1)	0.78	0.52–1.15	0.89	0.57–1.40
Q5 (>322.23)	93	(18.0)	103	(19.9)	0.76	0.51–1.13	0.83	0.53–1.31
**Vitamin B1 (thiamine)** (mg/day)								
Q1 (≤1.746)	138	(26.6)	104	(20.1)	1 (reference)		1 (reference)	
Q2 (1.747–1.970)	94	(18.2)	104	(20.1)	0.68 *	0.47–0.99	0.66	0.43–1.02
Q3 (1.971–2.226)	106	(20.5)	103	(19.9)	0.78	0.53–1.13	0.69	0.45–1.05
Q4 (2.227–2.589)	90	(17.4)	104	(20.1)	0.64 *	0.44–0.95	0.70	0.45–1.08
Q5 (>2.589)	90	(17.4)	103	(19.9)	0.64 *	0.43–0.95	0.64	0.41–1.01
**Vitamin B2 (riboflavine)** (mg/day)								
Q1 (≤1.895)	120	(22.2)	104	(20.1)	1 (reference)		1 (reference)	
Q2 (1.896–2.161)	101	(19.5)	104	(20.1)	0.84	0.57–1.23	0.76	0.49–1.16
Q3 (2.161–2.447)	101	(19.5)	103	(19.9)	0.85	0.59–1.24	0.80	0.52–1.23
Q4 (2.448–2.746)	95	(18.3)	104	(20.1)	0.79	0.54–1.16	0.80	0.52–1.24
Q5 (>2.746)	101	(19.5)	103	(19.9)	0.85	0.58–1.24	0.85	0.57–1.36
**Vitamin B3 (niacin)** (mg/day)								
Q1 (≤34.790)	137	(26.5)	104	(20.1)	1 (reference)		1 (reference)	
Q2 (34.791–39.035)	127	(24.5)	104	(20.1)	0.96	0.67–1.37	0.88	0.58–1.32
Q3 (39.036–42.430)	74	(14.3)	103	(19.9)	0.53 *	0.35–0.79	0.46 *	0.29–0.73
Q4 (42.431–47.830)	100	(19.3)	104	(20.1)	0.73	0.49–1.07	0.74	0.48–1.16
Q5 (>47.830)	80	(15.4)	103	(19.9)	0.58 *	0.39–0.86	0.59 *	0.37–0.86
**Vitamin B6 (pyridoxine)** (mg/day)								
Q1 (≤1.949)	134	(25.9)	104	(20.1)	1 (reference)		1 (reference)	
Q2 (1.950–2.257)	116	(23.4)	104	(20.1)	0.85	0.58–1.23	0.80	0.52–1.22
Q3 (2.258–2.508)	80	(15.4)	103	(19.9)	0.62 *	0.42–0.91	0.62 *	0.40–0.96
Q4 (2.509–2.858)	105	(20.3)	104	(20.1)	0.76	0.52–1.12	0.70	0.45–1.08
Q5 (>2.858)	83	(16)	103	(19.9)	0.62 *	0.42–0.93	0.69	0.43–1.08
**Vitamin B9 (folic)** (µg/day)								
Q1 (≤297.45)	138	(26.6)	104	(20.1)	1 (reference)		1 (reference)	
Q2 (297.46–348.78)	97	(18.7)	104	(20.1)	0.68 *	0.46–0.99	0.71	0.46–1.09
Q3 (348.79–412.94)	86	(16.6)	103	(19.9)	0.61 *	0.41–0.90	0.64	0.41–1.00
Q4 (412.95–491.91)	77	(14.9)	104	(20.1)	0.54 *	0.36–0.81	0.58 *	0.37–0.91
Q5 (>491.91)	120	(23.2)	103	(19.9)	0.87	0.59–1.27	0.88	0.57–1.35
								Continued
	**Cases (*n* 518)**		**Controls (*n* 518)**		**SGA**			
**Vitamin B12 (cyanocobalamin)** (µg/day)	***n***	**(%)**	***n***	**(%)**	**cOR**	**95% CI**	aOR *	**95% CI**
Q1 (≤6.633)	133	(25.7)	104	(20.1)	1 (reference)		1 (reference)	
Q2 (6.634–8.067)	109	(21)	104	(20.1)	0.81	0.56–1.18	0.91	0.59–1.39
Q3 (8.068–10.314)	102	(19.7)	103	(19.9)	0.79	0.54–1.15	0.80	0.52–1.24
Q4 (10.315–13.562)	83	(16)	104	(20.1)	0.61 *	0.41–0.91	0.61 *	0.39–0.95
Q5 (>13.562)	91	(17.6)	103	(19.9)	0.70	0.48–1.03	0.68	0.43–1.04

^a^ SGA: Small for Gestational Age; ^b^ cOR: Crude odds ratio and confidence intervals (95% CI) ^c^ aOR: Adjusted odds ratio by energy intake, preeclampsia, education level, pre-pregnancy body mass index, smoking, weight gain per week during pregnancy, and previous preterm/LBW newborn; * Significant association.

## Supplementary Materials

The following are available online at www.mdpi.com/2072-6643/9/12/1277/s1, Table S1: Multivitamin/mineral supplements during pregnancy among healthy Spanish women (*n* = 1036).
