# Supplementary material for: Association between Vitamin Intake during Pregnancy and Risk of Small for Gestational Age
Source: Nutrients. 2017 Nov 23;9(12):1277. doi: 10.3390/nu9121277 (PMC5748728; doi:10.3390/nu9121277)
Supplement: Supplementary file 1 [file nutrients-09-01277-s001.pdf]

nutrients

MDPI

Supplementary: Association between Vitamin Intake during Pregnancy and Risk of Small for Gestational Age

Inmaculada Salcedo-Bellido [1,2,3], Juan Miguel Martínez-Galiano [2,4,5,6,*], Rocío Olmedo-Requena [1,2,3], Juan Mozas-Moreno [2,7], Aurora Bueno-Cavanillas [1,2,3], Jose J. Jimenez-Moleon [1,2,3] and Miguel Delgado-Rodríguez [2,8]

Table S1. Multivitamin/mineral supplements during pregnancy among healthy Spanish women (n = 1036).

	**Cases (n = 518)**		**Controls (n = 518)**		**SGA[a]**			
	n	(%)	n	(%)	cOR[b]	95% CI	aOR[c]	95% CI
No	400	77.2	396	76.4	1 (reference)		1 (reference)	
Yes	118	22.8	122	23.6	0.95	0.71-1.29	0.93	0.66-1.29

[a]SGA: Small for Gestational Age; [b]cOR: Crude odds ratio and confidence intervals (95% CI); [c]aOR: Adjusted odds ratio by energy intake, preeclampsia, education level, pre-pregnancy body mass index, smoking, weight gain per week during pregnancy, and previous preterm/LBW newborn and anemia during pregnancy.
